# Effects of Maternal Suicidal Ideation on Child Cognitive Development: A Longitudinal Analysis

**DOI:** 10.1007/s10461-020-02802-8

**Published:** 2020-01-29

**Authors:** H. Mebrahtu, L. Sherr, V. Simms, H. A. Weiss, A. M. Rehman, P. Ndlovu, F. M. Cowan

**Affiliations:** 1 Institute of Global Health, University College London, London, UK; 2 MRC Tropical Epidemiology Group, School of Hygiene and Tropical Medicine, London, UK; 3 World Education Inc./Bantwana (WEI/B), Harare, Zimbabwe; 4 Centre for Sexual Health HIV/AIDS Research (CeSHHAR) Zimbabwe, Harare, Zimbabwe; 5 Department of International Public Health, Liverpool School of Tropical Medicine, Liverpool, UK; 6 Institute of Global Health, University College London, Royal Free Hospital Campus, Rowland Hill St, London, NW3 2PF UK

**Keywords:** Maternal mental health, HIV/AIDS, Child development, Suicidal ideation, Africa

## Abstract

This study aimed to assess the association between suicidal ideation among mothers living with HIV in Zimbabwe and the cognitive development of their children. Participants were mother–child dyads recruited from two rural districts in Zimbabwe. Data were collected at baseline and 12 months follow-up. Suicidal ideation was assessed using item-10 from the Edinburgh postnatal depression scale. Mixed-effects linear regression was used to assess the association of child cognitive outcomes at follow-up (using the Mullen scales of early learning) with maternal suicidal ideation. Mothers with suicidal ideation at baseline (n = 171) tended to be younger, unmarried, experienced moderate to severe hunger, had elevated parental stress and depression symptoms compared with non-suicidal mothers (n = 391). At follow-up, emerging maternal suicidal ideation was associated with poorer child cognitive outcomes (adjusted mean difference − 6.1; 95% CI − 10.3 to − 1.8; p = 0.03). Suicidal ideation affects child cognitive development and should be addressed, particularly in HIV positive mothers.

## Introduction

Suicidal ideation may precede suicide planning and attempted suicide and is a distressing psychological phenomenon, indicative of low mood and poor quality of life [1]. Despite advances in HIV treatment, suicidality (which includes suicidal ideation, suicide attempts, and completed suicides), remains elevated for people living with HIV (PLWH) in comparison with their HIV negative counterparts [2]. Although suicidal ideation is not always an automatic determinant for suicidal behaviour, it is an important risk factor that requires early detection and a potential trigger for intervention [3]. Risk factors for suicidal ideation in PLWH in sub-Saharan Africa (SSA) include younger age [3, 4], being unmarried and depression [4, 5]. Suicidal ideation among PLWH has also been linked to other factors such as lack of social support [4, 6], fear of HIV status disclosure and stigmatization [7]. Poverty further exacerbates the mental health burden among PLWH particularly in resource limited settings [8]. Increased negative life events and the associated stress in addition to food insecurity all increased the risk of suicidality in studies from Uganda, Peru, and Ethiopia [9–11]. Another study investigating predictive models for suicidal ideation and attempted suicide among women living with HIV in the US found that AIDS diagnosis, physical or sexual abuse, unemployment, and children were all significant predictors of suicide ideation and attempts [12].

There is evidence in the literature that shows child development is affected by maternal mental health [13], with exposure to maternal depression and stress having a negative influence on child development and being associated with impaired cognitive performance [14–16]. Infants of depressed mothers tend to show fewer affectively positive facial expressions and vocalization, more withdrawal, less attentiveness to the mother, decreased activity level, greater fussiness, and overall less engagement with people and objects [17]. Studies of suicide ideation specifically show maternal suicidal ideation is associated with poorer mother–infant relationship, and consequently negatively impacts infant development [17, 18]. In the long term, the quality of the early mother–infant relationship appears to predict aspects of child development, such as diverse forms of psychopathology, behavioural problems, and disruptions in cognitive abilities [19, 20]. In addition to this, the literature shows clear evidence of developmental delay for children in the presence of HIV infection and HIV exposure [21–23].

Nonetheless, current literature lacks clarity on the relationship between maternal suicidal ideation or behaviour on the child’s cognitive development, specifically in the context of HIV and in low resource settings. There is an urgent need for more evidence from low- and middle-income countries investigating the impact of suicidal ideation for mothers living with HIV over a period of time on child cognitive outcomes, especially during the early stages of development. This study uses longitudinal data to investigate the association of maternal suicidal ideation and child cognitive outcome in Zimbabwe, where there is recorded high HIV infection rates as well as elevated mental health burdens generally [13, 24–27], and suicidal challenges specifically.

## Methods

Data were collected for the cluster-randomized controlled trial (The Child Health Initiative for Developmental Outcomes-CHIDO [PACTR201701001387209]) [28]. Details of the trial methods have been published previously [28]. In brief, the aim was to determine the effectiveness of a combined parenting and income-generating programme delivered to caregivers living with HIV and their children aged 0–24 months at recruitment, on global child development in Zimbabwe.

### Study Design and Sample

Mother–child dyads were recruited from catchment areas surrounding 30 clinics in 2 rural districts in Mashonaland East Province. Trial clinics were randomized to the CHIDO intervention or Zimbabwe Ministry of Health and Child Care standard of care, and participants were enrolled in the arm to which their clinic was randomized. Participants in the standard care arm received the recommended standard of care for HIV positive mothers and their HIV exposed or infected children. Participants enrolled in the trial were assessed at baseline and followed up for 12 months for re-assessment. This analysis was confined to biological mothers only and their children who completed both assessments. All participants were provided with full information and gave consent to participate in the study as well as consent for child participation.

### Measures

Socio-demographic information was collected on participant characteristics (age, marital status), and socio-economic factors (educational level, employment status, asset index score, and number of adults living in the household), using interviewer-administered questionnaires. All children had an HIV test at follow-up.

A subset of questions from the household food insecurity access scale [29] were used to categorize participants as living: (i) food secure (rarely worried about food access or quality), (ii) moderately food insecure (sometimes i.e. 3–10 times in the last month, worried about food access or quality), or (iii) severely food insecure (≥ 1 household member going to bed hungry or often worrying about food access or quality).

The Edinburgh postnatal depression scale (EPDS), a postpartum depression-screening questionnaire that has been validated for use in Zimbabwe [30, 31], was administered to participating mothers. The EPDS comprises 10 questions that generate scores ranging from 0 to 30. A cut-off point of (12) which indicates concern for referral was used at baseline [30, 32]. The EPDS score was further categorized into none or minimal (EPDS scores 0–6), mild (EPDS scores 7–13), moderate (EPDS scores 14–19) and severe depression (EPDS scores 20–30) [33]; assessing the severity of maternal depressive symptoms for this population. The suicidal ideation item (item-10) was excluded from the total score.

Suicidal ideation was measured as thoughts of self-harm during screening using a self-reported questionnaire based on the EPDS [30, 31]. The EPDS scale contains a specific target item (item-10 “The thought of harming myself has occurred to me”) which assesses suicidal ideation [34–36] with good sensitivity (77%) and specificity (92%) according to previous studies in South Africa [2, 37]. Those responding “Yes, quite often”, “Sometimes” and “Hardly ever” in the past week were coded as experiencing suicidal ideation, whereas those to respond “Never” were coded as not experiencing suicidal ideation. Longitudinal data was utilised to categorise suicidal ideation over time into four groups. Women who did not report suicidal ideation at both baseline and 12 months follow-up were grouped as non-suicidal. Women reporting suicidal ideation at baseline but not at 12 months follow-up were grouped as improving and women who did not experience suicidal ideation at baseline but did at 12 months were referred to as the emerging suicidal ideation group. Whereas women who experienced suicidal ideation at both baseline and 12 months follow-up were marked as the chronic suicidal ideation group.

Parental stress index-short form (PSI-SF), a self-completed screening tool used for identifying different types of stress associated with parenting, was administered to participants [38]. Common mental disorders (CMDs) were assessed using the locally developed and validated Shona symptom questionnaire (SSQ)-8 [39]. The short form is derived from the longer SSQ-14 version. Scores range from 0 to 8, and scores ≥ 6 were used as a cut-off point for CMD symptoms.

Child cognitive development was assessed using the Mullen scales of early learning [40, 41]. The Mullen scale is a comprehensive measure that assesses a child’s abilities in five developmental domains from birth through 68 months: gross motor skills, visual reception, fine motor skills, receptive language, and expressive language [40]. The Mullen scales were administered to all children by trained assessors in a standardized format at enrolment and 12 months later [28]. The number of assessors was kept to a minimum to maximize the reliability of measurement. Test scores were transformed into an age-standardized T-score, using a US reference population as there was no local Zimbabwean reference population on this index. Four components—the fine motor, expressive language, receptive language, and visual perception scales—were combined to produce the age-standardized early learning composite (ELC) score of general cognitive functioning. The gross motor scale was not included in the ELC score and was used separately [40, 42].

### Data Analysis

To compare the characteristics of participants by suicidal ideation, descriptive analyses were used to summarise the baseline characteristics. Logistic regression was used to identify risk factors associated with suicidal ideation at baseline and was reported using odds ratio (OR) and 95% confidence intervals (95% CIs).

Data were pooled for this analysis as there was no evidence of a difference in child cognitive outcomes by trial arm. Mixed-effects linear regression was used to compare child cognitive outcomes by maternal suicidal ideation over 12 months. Mean children’s cognitive scores at follow-up by mother’s suicidal ideation categories were presented as adjusted mean differences (aMDs). Confounding variables associated with both exposure (maternal suicidal ideations) and outcome (child cognitive scores) in bivariate analyses (at p < 0.2) were included in the multivariable model. Clustering by study sites was accounted for by incorporating a random effect for clinic in all models. A priori adjustments included baseline child Mullen scores, mother’s age and the code for the person conducting Mullen assessments. All analyses were conducted using STATA v.15.1 (StataCorp LP, College Station, Texas, USA).

### Ethical Approval

The trial has been approved by the Medical Research Council of Zimbabwe (MRCZ/A/1943), University College London (6789/002) and the London School of Hygiene and Tropical Medicine (9912).

## Results

### Sample Characteristics at Baseline

The prevalence of suicidal ideation at baseline by demographic, socioeconomic, reproductive and mental health characteristics is shown in Table 1.

**Table 1:** Maternal demographic, socioeconomic, reproductive, mental health characteristics and child cognitive development by suicidal ideation at baseline

	Non-suicidal (n = 391)	Suicidal (n = 171)	Total (n = 562)	Test, p-value
Trial arm, n (%)				Chi^2^ = 2.28, p = 0.13
Intervention	181 (46.3)	91 (53.2)	272 (48.4)	
Control	210 (53.7)	80 (46.8)	290 (51.6)	
Age (years), mean (SD)	31.9 (6.3)	30.7 (6.2)	31.5 (6.3)	T = 1.97, p = 0.05
Education level (completed secondary school and above), n (%)	218 (55.8)	83 (48.5)	301 (53.6)	Chi^2^ = 2.49, p = 0.11
Relationship status^a^, n (%)				Chi^2^ = 31.98, p < 0.01
Married	335 (85.7)	112 (65.9)	447 (79.7)	
Divorced/separated	33 (8.4)	41 (24.1)	74 (13.2)	
Widowed	14 (3.6)	13 (7.7)	27 (4.8)	
Never been married	9 (2.3)	4 (2.4)	13 (2.3)	
Employment status (yes-employed), n (%)	146 (37.3)	60 (35.1)	206 (36.7)	Chi^2^ = 0.26, p = 0.61
Household size (number of people living under the same roof), mean (SD)	5.1 (1.6)	5.4 (1.9)	5.2 (1.7)	T = − 2.35, p = 0.02
Hunger scales, n (%)				Chi^2^ = 27.05, p < 0.01
Little to no hunger	271 (69.3)	79 (46.2)	350 (62.3)	
Moderate to severe hunger	120 (30.7)	92 (53.8)	212 (37.7)	
Asset Index score (terciles)^b^, n (%)				Chi^2^ = 3.52, p = 0.17
Low	126 (32.2)	63 (36.8)	189 (33.6)	
Middle	126 (32.2)	61 (35.7)	187 (33.3)	
High	139 (35.6)	47 (27.5)	186 (33.1)	
Tested for HIV, n (%)				Chi^2^ = 0.94, p = 0.33
Before pregnancy	214 (54.7)	86 (50.3)	300 (53.4)	
During or following pregnancy	177 (45.3)	85 (49.7)	262 (46.6)	
Child age (months), mean (SD)	11.8 (6.5)	12.3 (6.5)	11.9 (6.5)	T = − 0.87, p = 0.38
HIV status of child, n (%)				
HIV positive	8 (2.0)	7 (4.1)	15 (2.7)	–
HIV exposed and negative	329 (84.1)	149 (87.1)	478 (85.1)	–
HIV exposed, status unknown	54 (13.8)	15 (8.8)	69 (12.3)	–
Child cognitive development (Mullen scales), mean (SD)				
Expressive language	53.2 (10.6)	52.3 (11.0)	52.9 (10.7)	T = 0.97, p = 0.33
Receptive language	47.7 (11.3)	47.7 (12.0)	47.7 (11.5)	T = − 0.02, p = 0.99
Fine motor	50.9 (11.0)	50.5 (12.3)	50.8 (11.4)	T = 0.31, p = 0.76
Gross motor	50.9 (10.7)	49.7 (11.3)	50.5 (10.9)	T = 1.20, p = 0.23
Visual reception	53.3 (12.2)	52.7 (13.8)	53.1 (12.7)	T = 0.49, p = 0.62
Early learning composite score	102.7 (17.1)	102.0 (19.7)	102.5 (17.9)	T = 0.46, p = 0.65
Parental stress index, mean (SD)				
Parental distress	30.1 (6.9)	36.1 (7.4)	31.9 (7.6)	T = − 9.22, p < 0.01
Difficult child	27.6 (6.3)	29.7 (7.1)	28.2 (6.6)	T = − 3.66, p < 0.01
Parent–child dysfunction	24.0 (5.8)	26.1 (6.4)	24.6 (6.0)	T = − 3.81, p < 0.01
Total stress score	81.8 (15.0)	91.9 (17.0)	84.8 (16.3)	T = − 6.97, p < 0.01
Maternal depression scores (EPDS), mean (SD)	9.3 (5.7)	14.6 (5.0)	10.9 (6.0)	T = − 10.48, p < 0.01
Maternal depression scores-EPDS, n (%)				T = 88.94, p < 0.01
None	139 (35.6)	12 (7.0)	151 (26.9)	
Mild	149 (38.1)	55 (32.2)	204 (36.3)	
Moderate	91 (23.3)	72 (42.1)	163 (29.0)	
Severe	12 (3.1)	32 (18.7)	44 (7.8)	
SSQ-8, mean (SD)	3.6 (2.3)	6.3 (1.7)	4.4 (2.5)	T = − 13.35, p < 0.01
SSQ-8 (using ≥ 6 as cut off), n (%)				Chi^2^ = 93.00, p < 0.01
No CMD symptoms (scores 0–5)	286 (73.2)	51 (29.8)	337 (60.0)	
CMD symptoms (scores 6–8)	105 (26.9)	120 (70.2)	225 (40.0)	

*EPDS* the Edinburgh postnatal depression scale, *SSQ* Shona symptom questionnaire, *CMD* common mental disorder

^a^Relationship status variable was recoded to married/not married during analysis

^b^Several indicators were used for generating the asset index score including participants education level, number of people living in the same household, source of drinking water, employment status of mother and partner (if they had one), amount of money earned each month and a range of household items (such as electricity, refrigerator, bicycle, cell phone, television etc.)

At baseline, from the 574 participants enrolled, all 562 biological mothers completed the mental health assessments (the remaining 12 were other primary caregivers). The mean age of the mothers was 31.5 years (SD = 6.3), over half (53.6%) had secondary school and above level of education, over three quarters were married (79.7%), and 36.7% reported being formally or informally employed. The mean household size was 5.2 (SD = 1.7), and 37.7% of the households reported experiencing moderate to severe hunger. Over half of the women (55.5%) were diagnosed with HIV before their pregnancy and were aware of their status prior to conception. HIV status was ascertained for 493 children at follow-up and of these 15 (3.0%) were HIV positive.

There was no evidence of differences by trial arm in baseline prevalence of suicidal ideation among HIV positive mothers (53.2% suicidal ideation in the intervention arm vs. 46.8% control arm; p = 0.13). Suicidal ideation was associated with mother’s age, marital status, household size, food insecurity, parental stress and depression symptoms (Table 1). Mothers with suicidal ideation were likely to be slightly younger (mean age 30.7 vs. 31.9; p = 0.05), unmarried (34.2% vs. 14.3%; p < 0.01), lived in households that experience moderate to severe hunger (53.8% vs. 30.7%; p < 0.01), have elevated parental stress (PSI-SF mean − 91.9 vs. 81.8; p < 0.01) and depression symptoms (EPDS mean − 14.6 vs. 9.3; p < 0.01) compared to the non-suicidal group. Mothers with suicidal ideation were also more likely to experience severe depression (OR 16.8; 95% CI 4.5 to 62.3; p < 0.01) and CMD symptoms (OR 5.5; 95% CI 2.9 to 10.5; p < 0.01) compared to the non-suicidal group (Table 2).

**Table 2:** Logistic regression model of risk factors associated with maternal suicidal ideation at baseline (n = 562)

	N	Odds ratio (95% CI)	p value
Age group (years)			
16–19	16	Ref	
20–30	216	4.2 (0.8 to 21.2)	0.08
31–40	282	2.1 (0.4 to 10.7)	0.35
41–49	48	1.1 (0.2 to 6.4)	0.92
50–70	–	–	–
Married			
No	115	Ref	
Yes	447	0.2 (0.1 to 0.4)	< 0.01
Number of people living in the household			
2–3	83	Ref	
4–6	375	1.4 (0.6 to 3.2)	0.40
7–9	95	1.4 (0.5 to 3.7)	0.55
10–13	9	3.8 (0.5 to 28.1)	0.20
Hunger scale			
Little to no hunger	350	Ref	
Severe to moderate hunger	212	3.2 (1.9 to 5.6)	< 0.01
Maternal depression (EPDS category)			
None	151	Ref	
Mild	204	2.5 (1.1 to 5.8)	0.04
Moderate	163	2.7 (1.1 to 6.7)	0.03
Severe	44	16.8 (4.5 to 62.3)	< 0.01
SSQ-8			
No CMD symptoms	337	Ref	
CMD symptoms	225	5.5 (2.9 to 10.5)	< 0.01

*OR* odds ratio, *EPDS* the Edinburgh postnatal depression scale, *SSQ* Shona symptom questionnaire, *CMD* common mental disorder

### Suicidal Ideation over Time

After 12 months 514 participants completed a follow-up survey, but only 485 (86.3%) pairs were both completed by the child’s biological mother. Of the 485 mothers, 86 (17.7%) reported suicidal ideation at both timelines (chronic), 66 (13.6%) had suicidal ideation at baseline but not at 12 months follow-up (improvers); 46 (9.5%) mothers reported emerging suicidal ideation at 12 months only. The largest group (n = 287; 59.2%) did not report suicidal ideation at either time points.

Table 3 shows Mullen cognitive development scores by suicidal ideation group. Children of mothers with emerging suicidal ideation had consistently lower mean T-scores across all child development domains, compared to the other groups.

**Table 3:** Mullen T-scores of children at 12 months follow-up by maternal suicidal ideation categories

Mullen Scales (T-scores)	Not suicidal at both baseline and follow-up, n = 287	Suicidal ideation at baseline only, n = 66	Suicidal ideation at follow-up only, n = 46	Suicidal at both baseline and follow-up, n = 86
	Mean (SD)	Mean (SD)	Mean (SD)	Mean (SD)
Expressive language	45.4 (9.2)	46.1 (9.6)	42.2 (8.3)	44.3 (10.0)
Receptive language	46.1 (9.6)	46.8 (9.4)	41.3 (12.2)	44.1 (10.6)
Fine motor	41.9 (11.2)	42.3 (10.9)	37.3 (9.5)	40.5 (9.8)
Gross motor^a^	49.9 (11.3)	50.7 (11.3)	44.0 (11.8)	48.3 (11.4)
Visual reception	42.5 (10.9)	42.7 (11.3)	37.2 (10.7)	41.7 (11.2)
Early learning composite score	88.7 (15.2)	89.4 (14.3)	80.5 (14.9)	86.2 (15.8)

^a^Only measured in children aged < 36 months at follow-up (n = 397)

In multivariable analysis, children of the women with emerging suicidal ideation had lower receptive language (aMD − 4.2; 95% CI − 7.2 to − 1.2; p = 0.02), visual reception (aMD − 4.4; 95% CI − 7.6 to − 1.2; p = 0.04), and overall composite score (aMD − 6.1; 95% CI − 10.3 to − 1.8; p = 0.03) compared to the children of the reference group (Table 4). There was also an evidence of difference in the children’s gross motor functioning by maternal suicidal ideation categories (aMD − 5.2; 95% CI − 8.9 to − 1.5; p = 0.01).

**Table 4:** Regression models of child cognitive outcomes and maternal suicidal ideation

	No suicidal ideation (n = 287)	Suicidal ideation at baseline only (n = 66)	Suicidal ideation at follow-up only (n = 46)	Suicidal at both baseline and follow-up (n = 86)	p value*
		Adjusted mean difference (95% CI)			
Expressive language	Ref	1.23 (− 1.20 to 3.67)	− 2.27 (− 5.13 to 0.59)	− 0.26 (− 2.47 to 1.95)	0.25
Receptive language	Ref	1.12 (− 1.45 to 3.69)	− 4.18 (− 7.19 to − 1.16)	− 1.49 (− 3.81 to 0.82)	0.02
Fine motor	Ref	0.70 (− 2.10 to 3.49)	− 3.45 (− 6.76 to − 0.15)	− 0.87 (− 3.40 to 1.65)	0.16
Gross motor^a^	Ref	2.16 (− 1.08 to 5.40)	− 5.18 (− 8.91 to − 1.46)	0.03 (− 3.08 to 3.14)	0.01
Visual reception	Ref	0.71 (− 1.99 to 3.41)	− 4.37 (− 7.56 to − 1.18)	− 0.37 (− 2.80 to 2.07)	0.04
Early learning composite score	Ref	1.35 (− 2.28 to 4.98)	− 6.05 (− 10.34 to − 1.77)	− 0.98 (− 4.26 to 2.31)	0.03

*Model adjusted for baseline Mullen scores, mother’s age, clustering of trial sites and examiner

^a^Only measured in children aged < 36 months at follow-up (n = 397)

## Discussion

This study aimed to explore maternal suicidal ideation in the presence of HIV and the association between such ideation over time with child cognitive development. Suicidal ideation in this group was high. Almost a third of the mothers reported suicidal ideation at baseline, and this was reduced to 27.2% at endline. Mothers with suicidal ideation at baseline were more likely to be divorced or separated and suffer from elevated stress and depressive symptoms. In addition, household food insecurity levels were high in mothers with suicidal ideation. The results show evidence of an association between maternal suicidal ideation over time and child cognitive performance for this HIV affected sample. Children’s gross motor, visual and receptive language abilities were the developmental domains most closely associated with maternal suicidal ideation. Additionally, children of mothers with emerging suicidal ideation tended to have lower cognitive scores across the developmental domains compared to the other groups.

The results here support previous research in which suicidal ideation was associated with marital status, food insecurity, maternal depression and stress [2, 4–6]. The lack of a support system or household food security may all contribute to the mother’s mental well-being and the likelihood of suicidal thoughts or suicidal behaviour manifestation. Alternatively, suicidal ideation may mitigate against relationship formation and affect the ability to gather resources ensuring food security. Other studies in rural areas report being single or widowed, and being older in age is associated with increased food insecurity in PLWH [43]. Considering this was a rural population, there could also be high levels of food insecurity simply due to higher levels of poverty compared to urban areas, or limited availability of plots of land per family [44]. Suicidal ideation may be a result of the burden of these stressors and contemplating suicide a pathway to escape from them [45]. The findings suggest a clustering of negative life events such as HIV, and other life stressors result in elevated and chronic suicidal ideation. Although the direction of the effect between suicidal ideation and the factors discussed above cannot be definitively established, these associations point to both warning signs and intervention opportunities.

Similar to previous studies, maternal mental health was negatively associated with child development in the language and visual domains [13, 14]. It may be that mental health burdens and stress distract mothers from parenting and child stimulation reducing visual and verbal interactions and may affect the crucial child development steps. Deteriorating maternal mental health (i.e. emerging suicidal ideation) in particular seems to affect child cognition overall. Interestingly, the children of the improvers and reference group (i.e. no suicidal ideation) had similar scores on most of the developmental domains. Therefore, improving mental wellbeing or interruption of suicidal thoughts in mothers may potentially feed into improved maternal mental health and child outcomes over time. Furthermore, this study examines longitudinal changes in maternal suicidal ideation over 12 months for women living with HIV. The use of longitudinal analysis is pivotal in order to further understand the relationship between emerging maternal mental health issues and child development.

Some of the strengths of this study include a large sample size and utilization of previously validated assessment tools in similar settings such as the EPDS and SSQ-8. A study by Paris et al. (2009) examined the relationship between suicidality and mother–infant interactions using cross-sectional data [18]. Other studies in southern Africa have examined predictors of suicidal ideation in pregnancy/postpartum using cross-sectional data [2, 11, 46]. This study is the only one to our knowledge that investigates the impact of maternal suicidal ideation on child’s cognitive development over time. This study adds useful insight to the literature on the role of maternal mental health on child development in the SSA context and highlights the impact of emerging maternal suicidal ideation on child cognitive development. The results here also provide valuable information on the characteristics of HIV positive mothers at risk of suicidal ideation in rural settings. Future studies examining maternal suicidal ideation and child development should utilize these socioeconomic predictors to identify the mothers who are at high risk for depression and suicidal ideation in similar settings. Findings from this study can also aid the development of suicide prevention and synergic intervention measures for HIV positive mothers and their children in a similar context.

Some limitations need to be taken into account. The data were collected in a trial and although there was no evidence of differences between trial arms, the changes over time may not reflect a true field situation. Although follow-up was in excess of 86%, there may have been some attrition associated with key variables. Additionally, the Mullen scales use a USA reference group which is not ideal given the setting of the study in Zimbabwe. Locally validated scales and reference groups would have been preferable.

Despite these limitations, the data clearly show the high prevalence of suicidal ideation in HIV positive mothers, with close to a quarter of the sample recording suicidal ideation at baseline. Although this rate goes down over time, the rates at follow-up are still high and indicate an unmet mental health need. The results also show that maternal suicidal ideation can negatively affect child development domains, specifically their verbal and visual skills. Emerging maternal suicidal ideation, in particular, seems to affect child cognition. Early child development is critical for later achievement. Given that a proportion of these children were HIV positive and all were HIV exposed there is already a concern for their cognitive development. This seems to be compounded by maternal suicidal ideation. Early identification and treatment of HIV positive mothers with suicidal ideation, can help improve their mental health, adherence to treatment and overall quality of life [24] and consequently result in long term gains for their children’s development.
